# Targeting aerobic glycolysis by dichloroacetate improves Newcastle disease virus-mediated viro-immunotherapy in hepatocellular carcinoma

**DOI:** 10.1038/s41416-019-0639-7

**Published:** 2019-12-10

**Authors:** Gang Meng, Binghua Li, Anxian Chen, Meihong Zheng, Tiancheng Xu, Hailin Zhang, Jie Dong, Junhua Wu, Decai Yu, Jiwu Wei

**Affiliations:** 1 Department of Hepatobiliary Surgery, the Affiliated Drum Tower Hospital, Medical School of Nanjing University, Nanjing, 210008 China; 2 Jiangsu Key Laboratory of Molecular Medicine, Medical School of Nanjing University, Nanjing, 210093 China

**Keywords:** Cancer immunotherapy, Cancer metabolism

## Abstract

**Background:**

Oncolytic viro-immunotherapy holds promise for cancer treatment. While immune activation can be robustly triggered by oncolytic viruses, negative feedback is often upregulated in the tumour microenvironment (TME). Lactate accumulation, signal transducer and activator of transcription 3 (STAT3) activation, indoleamine 2,3-dioxygenase 1 (IDO1) expression, and myeloid-derived suppressor cell (MDSC) infiltration coordinate to shape the immunosuppressive TME.

**Methods:**

Representative hepatocellular carcinoma (HCC) cell lines and HCC-bearing mice were treated with oncolytic Newcastle disease virus (NDV), alone or in combination with dichloroacetate (DCA, a pyruvate dehydrogenase kinase (PDK) inhibitor).

**Results:**

We found that infection with oncolytic NDV led to significant induction of the aforementioned suppressive factors. Interestingly, DCA significantly reduced lactate release, STAT3 activation, IDO1 upregulation, and MDSC infiltration in NDV-treated HCC. Consequently, DCA significantly enhanced the antitumour immune responses, leading to improved antitumour efficacy and prolonged survival in mouse models of ascitic and subcutaneous HCC. Furthermore, DCA increased NDV replication in a PDK-1-dependent manner in HCC.

**Conclusions:**

Targeting aerobic glycolysis by DCA improves NDV-mediated viro-immunotherapy in HCC by mitigating immune negative feedback and promoting viral replication. These findings provide a rationale for targeting reprogrammed metabolism together with oncolytic virus-mediated viro-immunotherapy for HCC treatment.

## Background

Hepatocellular carcinoma (HCC) is one of the leading causes of cancer-related deaths worldwide.^1^ There is an urgent need for novel strategies to enhance treatment responses in patients with HCC. Oncolytic viruses (OVs) are therapeutically useful viruses that preferentially infect and damage cancerous tissues without harming normal tissues.^2^ Genetically engineered human herpes simplex virus 1 (HSV-1) expressing granulocyte-macrophage colony-stimulating factor (GM-CSF; T-VEC, also known as OncoVEX^GM-CSF^) has been approved by the US Food and Drug Administration for use in melanoma patients.^3^ Oncolytic viro-immunotherapy is an attractive strategy for HCC. The genetically engineered oncolytic vaccinia virus expressing GM-CSF (Pexa-Vec or JX-594) induced antitumour immunity and significantly prolonged survival in a dose-dependent manner in HCC clinical trials.^4^

Newcastle disease virus (NDV) is a member of the *Avulavirus* genus in the *Paramyxoviridae* family. It has a small genome (15.2 kb, negative single-stranded RNA) that encodes six structural genes. The oncolytic properties of NDV in tumour cells were first reported ~50 years ago.^5^ Previous studies suggested that the selectivity of NDV for tumour cells depends on its interactions with antiviral type I interferons (IFNs) and the apoptotic pathway.^6,7^ Several preclinical and clinical trials have shown that NDV is effective in treatment of tumours without affecting normal cells.^8,9^ Furthermore, there is increasing evidence that, in addition to its direct oncolytic effect, NDV activates the immune system to trigger tumour rejection via a specific or nonspecific antitumour immune response.^10,11^ Localised therapy with oncolytic NDV induces inflammatory immune cell infiltrates (including natural killer (NK) 1.1^+^, CD3^+^CD8^+^, CD11b^+^ lymphocytes, and monocytes), both in injected tumours and in distant tumours without distant virus spread, such that the tumours become susceptible to systemic treatment with immunotherapies.^12^ The therapeutic efficacy of NDV is dependent on CD8^+^ T cells, NK cells, and type I IFNs, but not CD4^+^ lymphocytes.^12,13^

Most tumour cells are characterised by high rates of aerobic glycolysis (Warburg effect).^14^ Aerobic glycolysis plays an important role in shaping the immunosuppressive tumour microenvironment (TME). Notably, glycolysis within tumour cells has been shown to cause depletion of extracellular glucose, which restricts glucose availability to T cells. This reduced glucose availability causes suppression of glycolytic metabolism within T cells, which is associated with reduced proliferation and effector function.^15^ In addition, the accumulation of lactate due to glycolysis in the TME severely impacts the functional properties of T cells and NK cells.^16,17^ Inhibition of glycolysis has been reported to enhance the antitumour T-cell response in the TME.^18,19^ Coincidentally, viral infection, such as with measles virus, adenovirus, human immunodeficiency virus, or human cytomegalovirus, dramatically shifts host cellular glucose metabolism to high-level glycolysis,^20–23^ which could be an obstacle for oncolytic viro-immunotherapy. It is not yet known whether NDV infection also increases glycolysis.

The interleukin-6/Janus kinase/signal transducer and activator of transcription 3 (IL-6/JAK/STAT3) inflammation pathway, indoleamine 2,3-dioxygenase-1 (IDO1)-mediated tryptophan metabolism, and myeloid-derived suppressor cells (MDSCs) play a crucial role in the restriction of effective antitumour immunity.^24–26^ These suppressive factors can be upregulated by immune activation, including OVs, and may restrict OV-mediated antitumour immune responses.^25–28^ Hence, the restriction of such negative feedback is crucial for the improvement of oncolytic viro-immunotherapy.^29^

The orphan drug dichloroacetate (DCA) is a small-molecule inhibitor of mitochondrial pyruvate dehydrogenase kinase (PDK). PDK inhibition leads to the reactivation of pyruvate dehydrogenase in the mitochondria, where it increases the ratio of glucose oxidation to glycolysis.^30^ DCA has been used to treat human hereditary mitochondrial metabolic diseases and lactic acidosis for more than 30 years. It has also been evaluated in a variety of pre-clinical cancer models and clinical trials, including prostate cancer, glioblastoma, and non-small cell lung cancer.^30,31^ Importantly, a previous study showed that DCA improved the antitumour activity of T cells by reducing lactate-mediated immunosuppression.^32^

In this study, we investigated immune status alterations in the HCC TME by NDV infection. We also investigated the function of DCA in NDV-mediated immunotherapy in HCC. These findings provide a rationale for combinatorial strategies in OV-mediated immunotherapy.

## Methods

The materials and methods described here have been reported previously^29^ and are outlined briefly below.

### Cell culture and reagents

The human HCC cell line HCCLM3 and mouse HCC cell line H22 were obtained from the China Center for Type Culture Collection, the mouse HCC cell line Hepa1–6 was obtained from Cell Bank of Type Culture Collection Chinese Academy of Sciences, authenticated by short tandem repeat (STR) analysis, and tested for mycoplasma contamination. HCCLM3 (abbreviated LM3) and Hepa1–6 cells were cultured in high glucose (4.5 g/L) Dulbecco’s modified Eagle’s medium (DMEM) and H22 cells were cultured in Roswell Park Memorial Institute 1640 Medium (RPMI 1640) supplemented with 10% foetal bovine serum, 2 mM l-glutamine, 100 units/mL penicillin, and 0.1 mg/mL streptomycin (all from Thermo Fisher Scientific, Gibco, Grand Island, NY, USA). All cells were maintained in a humidified incubator with an atmosphere containing 5% CO_2_ at 37 °C.

The following reagents were used: sodium dichloroacetate (#347795; Sigma-Aldrich, St. Louis, MO, USA), trypan blue (#ST798; Beyotime Biotechnology, Shanghai, China), and 3-(4,5-dimethyl-2-thiazolyl)-2,5-diphenyl-2H-tetrazolium bromide (MTT) (#M2128; Sigma-Aldrich).

### NDV propagation, viral titres, and infection

The NDV La Sota strain was a gift from Prof. Y. Wang (Jiangsu Academy of Agricultural Sciences, China). It was propagated in 9-day-old specific pathogen-free (SPF) embryonated chicken eggs from seed virus, harvested from allantoic fluid, and purified by centrifugation at 1000 × *g* for 10 min. The viral particles in the supernatant were harvested and cryopreserved at −80 °C. Viral titres were determined by plaque assay. Briefly, samples were serially diluted, and 100 µL of each serial dilution was added per well to Vero cells in 12-well plates. After 2 h of adsorption, cells were overlaid with DMEM (containing 2 µg/mL TPCK-treated trypsin, 2% FBS, and 0.8% low melting point agarose) and then incubated at 37 °C for 4 days. The cells were then fixed with 4% paraformaldehyde solution and stained with 0.5% neutral red solution for observation of plaques.

Tumour cells were washed once with phosphate-buffered saline (PBS) and infected with NDV in OptiMEM (Thermo Fisher Scientific, Gibco) at the indicated multiplicity of infection (MOI) for 2 h, and complete medium was added to each well.

### Cytotoxic effects

#### MTT cell viability assay

Cells were seeded in 96-well plates and treated with DCA and/or NDV at the indicated doses. Cell viability was determined after 48 h of incubation by adding 100 μL of MTT solution (1 mg/mL) to each well. Following 4 h incubation at 37 °C, the MTT solution was aspirated, 150 μL of isopropanol was added to solubilise the formazan, and the plates were shaken for 15 min. The absorbance at 570 nm was recorded using a SpectraMax^®^ M3 Multi-Mode Microplate Reader (Molecular Devices, Sunnyvale, CA, USA).

#### Trypan blue exclusion

Cells were harvested using trypsin-EDTA (0.25%) solution (Thermo Fisher Scientific, Gibco) and stained with trypan blue; viability was determined using trypan blue exclusion assays with a Countstar Automated Cell Counter (Inno-Alliance Biotech Inc., Wilmington, DE, USA).

### Short interfering RNA transfection

Short interfering RNA (siRNA) (200 nM) coupled with Lipofectamine™ 2000 (#11668019; Thermo Fisher Scientific, Invitrogen) was used for transfection on a 6- or 12-well plate according to the manufacturer’s instructions. The sequences of siRNA targeting PDK-1 and non-specific control siRNA are listed in the Supplementary Table. NDV infection or drug treatment was performed at 4 h after siRNA transfection in PDK-1-silencing experiments.

### Glucose uptake and lactate release assay

The glucose concentration in culture supernatants was determined using a glucose colorimetric assay kit (#361500; Shanghai Rongsheng Biotech, Shanghai, China) according to the manufacturer’s instructions and quantified by measuring the absorption at 450 nm. Glucose uptake was characterised as the original glucose concentration in the medium minus the detected glucose concentration in culture supernatants. Lactate generation was measured using a lactate colorimetric assay kit (#A019-2; Nanjing Jiancheng Bioengineering Institute, Nanjing, China) according to the manufacturer’s instructions. Briefly, NAD^+^ was added to the media, wherein it is converted to NADH stoichiometrically by lactate. The level of lactate was quantified colorimetrically at 530 nm.

### Western blot

Cells were lysed in RIPA buffer (#P0013C; Beyotime) containing protease inhibitor tablets (#05892791001; Roche, Indianapolis, IN, USA) and the protein concentrations were determined using an Enhanced BCA Protein Assay Kit (#P0010, Beyotime). Equal amounts of protein were separated by sodium dodecyl sulphate-polyacrylamide gel electrophoresis (SDS-PAGE) and transferred electrophoretically to polyvinylidene fluoride membranes (#03010040001; Roche). After blocking in 5% non-fat milk in Tris-buffered saline, the membranes were incubated with specific primary antibodies followed by appropriate horseradish peroxidase (HRP)-conjugated secondary antibodies. Signals were detected using enhanced chemiluminescence reagent (#WBKLS0500; Millipore, Billerica, MA, USA) and analysed using a chemiluminescent imaging system (ChampChemi 610; Sage Creation Science, Beijing, China). Antibodies against the following proteins were used: GAPDH (#MB001; 1:5000; Bioworld, St. Louis Park, MN, USA), STAT3 (#9139; 1:1000; Cell Signaling Technology, Danvers, MA, USA), phospho-STAT3 (Tyr705) (D3A7) (#9145; 1:1000; Cell Signaling Technology), PDK-1 (#3820; 1:1000; Cell Signaling Technology), and IDO1 (#ab55305 & ab106134; 1:500; Abcam, Cambridge, UK). HRP-conjugated secondary antibodies (#31430 & 31460; 1:2000) were purchased from Thermo Fisher Scientific/Pierce.

### Quantitative RT-PCR

Total cellular RNA was extracted using TRIzol (#15596-026; Thermo Fisher Scientific, Invitrogen) and reverse-transcribed using PrimeScript™ RT Master Mix (#DRR036A, TaKaRa, Shiga, Japan). Quantitative PCR was performed using FastStart Universal SYBR^®^ Green Master Mix (#04913914001; Roche) on a ViiA™ 7 Real-Time PCR System (Applied Biosystems, Foster, CA, USA). Gene expression was calculated using the comparative Ct method and normalised to that of *GAPDH*. The primer sequences are listed in the Supplementary Table.

### Animal experiments and tumour models

Six-week-old male *C57BL/6* mice were obtained from the Model Animal Research Center of Nanjing University (Nanjing, China), and were maintained under SPF conditions (temperature 22 ± 2 °C, humidity 60 ± 10%, 12/12 h light/dark cycle) at Medical School of Nanjing University. All experimental procedures were approved by the Ethics Committee of the Affiliated Drum Tower Hospital of Nanjing University Medical School and animal welfares were closely monitored in accordance with the Guide for the Care and Use of Laboratory Animals of the National Institutes of Health.

The ascitic HCC model: tumours were implanted by intraperitoneal (i.p.) injection of 2 × 10^6^ H22 on day 0. H22-bearing mice were randomised into four different treatment groups (Untreated, DCA, NDV, and NDV-DCA) on day 2. From days 3 to 17, mice received 200 mg/kg DCA or an equal volume of sterile water intragastrically (i.g.) every day. On days 4, 5, 8, 9, 12, and 13, mice received an i.p. injection of 1 × 10^7^ plaque-forming units (pfu) of NDV per mouse in PBS in a total volume of 100 µL or an equal volume of PBS. On days 10 and 15, ascites samples (500 µL) were removed to determine cell number, NDV replication, antiviral gene expression, immune cell infiltration, IFN-γ ELISpot, IDO1 expression, and STAT3 phosphorylation. The body weight and behaviour of the mice was monitored every other day and survival was monitored every day. Mice were killed by cervical dislocation when their condition was assessed to be moribund.

The subcutaneous HCC model: mice received subcutaneous injection of 5 × 10^6^ Hepa1-6 cells of each. Hepa1-6-bearing mice were randomly assigned to four different treatment groups (Untreated, DCA, NDV, and NDV-DCA) on day 5. On days 7, 10, and 13, the mice received an intratumoural injection of 1 × 10^7^ pfu NDV per mouse, respectively. From day 7 to 20, mice received 200 mg/kg DCA or an equal volume of sterile water i.g. every day. Tumour volume was monitored every 2–3 days by calliper measurement and calculated by length × width × width/2. The body weight was monitored every other day and survival was monitored every day. Mice were killed by cervical dislocation when tumour volume reached 3 cm^3^, or when mice appeared moribund.

### Flow cytometry

For immune activation experiments in vivo, ascitic cells were harvested, washed twice with PBS, and incubated with the following antibodies: CD11b (clone M1/70, #557396), Gr-1 (clone RB6-8C5, #553128) (both from BD Biosciences, Franklin Lakes, NJ, USA), and isotype-specific antibodies (11-4714-41, 17-4031-81; Thermo Fisher Scientific, eBioscience). CD11b^+^Gr-1^+^ cells were defined as MDSCs. Samples were subjected to flow cytometry using a FACSCalibur instrument (BD Biosciences) and data were analysed using FlowJo software (ver. 7.6.5; Tree Star Inc., Ashland, OR, USA).

### IFN-γ enzyme-linked immunosorbent spot assay

The activation status of immune cells in ascites was evaluated using a Mouse IFN-γ EILSpot kit (3321-2AW-Plus; Mabtech, Nacka Strand, Sweden) according to the manufacturer’s protocol. Briefly, ascites cells were seeded in a 96-well plate coated with an IFN-γ capture antibody at a density of 2 × 10^5^ cells/well and incubated at 37 °C for 24 h in a humidified incubator with an atmosphere containing 5% CO_2_. Then, the cells were removed, the plate was washed five times with PBS, and a biotinylated anti-IFN-γ antibody was added; the plate was then incubated at room temperature for 2 h. Next, the plate was washed with PBS and streptavidin-ALP was added; then, the plate was incubated at room temperature for 1 h. Finally, BCIP/NBT-plus substrate was added until spots emerged, and the plate was washed with tap water to stop the reaction. The plate was analysed using an enzyme-linked immunosorbent spot (ELISpot) reader (Autoimmun Diagnostika GmbH, Strassberg, Germany) to enumerate spots, and spot activity was characterised as the weighted average of the spot size and intensity in a well.

### Statistical analyses

Data were analysed using two-tailed unpaired Student’s *t*-tests (for comparisons of two groups). Survival data were analysed using log-rank (Mantel-Cox) tests. Statistical analyses were conducted using Microsoft Excel (Microsoft Corp., Redmond, WA, USA) or Prism software (ver. 7.0; GraphPad Software, Inc., La Jolla, CA, USA).

## Results

### DCA reduces lactate release in NDV-treated HCC

**Fig. 1:**
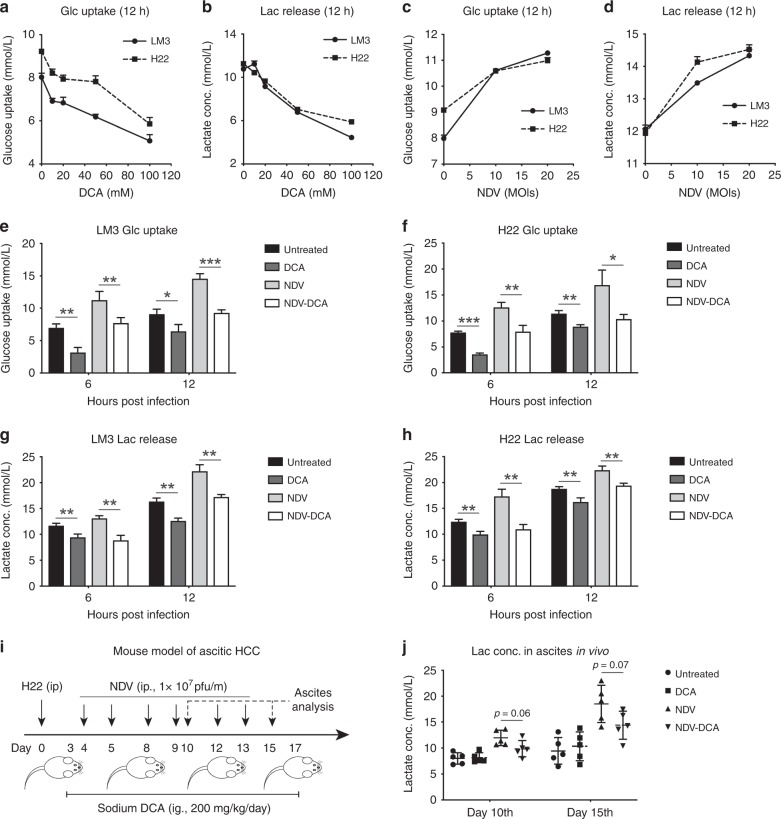
DCA reduces lactate release in NDV-treated HCC. **a**–**d** LM3 and H22 cells were treated with different dose of DCA for 12 h (**a**, **b**), or infected with NDV at different multiplicity of infection (MOI) for 12 h (**c**, **d**), and the concentrations of glucose and lactate in the media were measured. Means and standard deviations of quadruplicate samples are shown. **e**–**h** LM3 and H22 cells were infected with NDV (MOI = 10) in the presence or absence of DCA (20 mM) for 6 or 12 h. Glucose uptake (**e**, **f**) and lactate release (**g**, **h**) were measured. Means and standard deviations of quadruplicate samples are shown. **i**, **j** Mice received an i.p. injection of 2 × 10^6^ H22 cells. Four days later, they were injected i.p. with 1 × 10^7^ pfu NDV for six rounds at the indicated time points (*n* = 5 per group), with or without DCA (200 mg/kg, daily, i.g.) (**i**). Ascitic fluid was harvested after 10 or 15 days for lactate assay; relative lactate concentrations were quantified and compared with the untreated group at day 10 (**j**). Means and standard deviations of five mice are shown. *, *p* < 0.05; **, *p* < 0.01; ***, *p* < 0.001

First, we confirmed that DCA efficiently inhibited glycolysis in a dose-dependent manner in HCC cells (Fig. 1a, b). In addition, we measured the pH of medium containing a different concentration of sodium DCA up to 100 mM (Fig. S1). The pH of medium with 20 mM DCA is 7.40, which is acceptable since the normal blood pH is between 7.35 and 7.45. Therefore, 20 mM DCA was used in the rest of the study, which minimises any potential pH effects. Then, we showed that aerobic glycolysis was significantly upregulated in NDV-infected HCC cells, as determined by glucose uptake and lactate release (Fig. 1c, d); this could be significantly inhibited by DCA (Fig. 1e–h). Further investigation revealed that among the four PDK subtypes, only PDK-1 mRNA was upregulated by NDV infection (24 h post infection, Fig. S2), suggesting the importance of PDK-1 in NDV-induced high-rate glycolysis. In an ascitic HCC mouse model (Fig. 1i), NDV treatment resulted in an accumulation of lactate in ascites, and DCA partially reduced this accumulation (Fig. 1j). Figure S3 shows the half-maximal inhibitory concentration (IC_50_) and time kinetics of NDV and DCA in HCC cells, respectively. These results suggested that DCA blocks NDV-upregulated glucose metabolism in HCC.

### DCA mitigates NDV-induced STAT3 activation, IDO1 expression, and MDSC infiltration in HCC

**Fig. 2:**
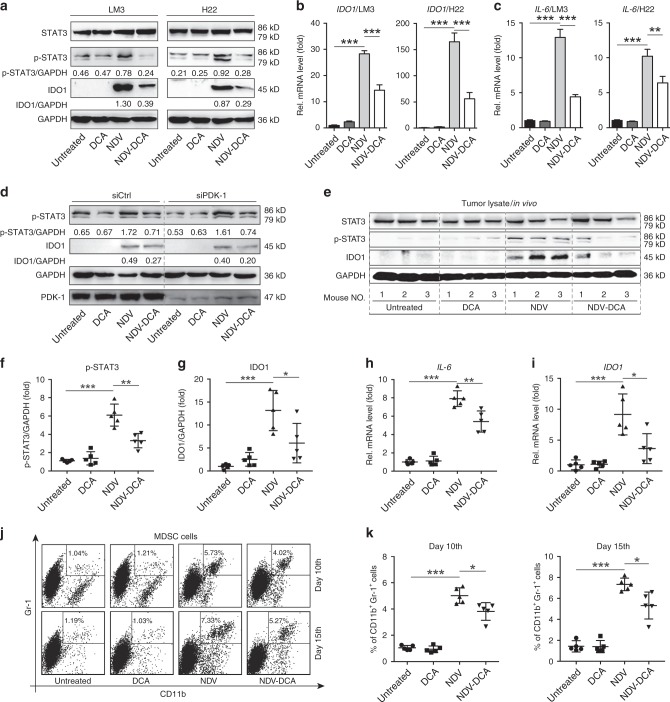
DCA mitigates NDV-induced STAT3 activation, IDO1 upregulation, and MDSC infiltration in vitro and in vivo. **a** LM3 and H22 cells were infected with NDV (MOI = 10) in the absence or presence of DCA (20 mM) for 24 h, then lysed; total STAT3, phosphorylated STAT3 (p-STAT3), and IDO1 protein expression levels were determined by western blotting. **b**, **c** Cells were infected with NDV (MOI = 10) in the absence or presence of DCA (20 mM) for 24 h, then harvested; *IDO1* (B) and *IL-6* (C) gene expression levels were determined by qPCR. Means and standard deviations of quadruplicates are shown. **d** LM3 cells were transfected with an siRNA targeting PDK-1 (siPDK-1) for 4 h followed by NDV infection (MOI = 10) in the absence or presence of DCA (20 mM) for 24 h, then cells were lysed; p-STAT3, IDO1, and PDK-1 protein expression levels were determined by western blotting. **e**–**i** Mice were treated as previously described (Fig. 1I), ascitic cells were harvested at day 15, and IDO1 and p-STAT3 protein expression levels (**e**) were determined by western blotting; *Il-6* (**h**) and *Ido1* (**i**) gene expression levels were quantified by qPCR. Representative blots (**e**), and the ratios of IDO1/GAPDH (**f**) and p-STAT3/GAPDH (**g**) are shown. **j**, **k** Ascitic cells were harvested after 10 or 15 days for flow cytometric analysis of myeloid-derived suppressor cells (MDSCs) (CD11b^+^ Gr-1^+^). Representative graphs (**j**) and statistical analysis (**k**) are shown. Means and standard deviations of five mice are shown. *, *p* < 0.05; **, *p* < 0.01; ***, *p* < 0.001

STAT3 activation, IDO1 upregulation, and MDSC infiltration contribute to tumour-associated immune tolerance. We found that NDV infection also activated the IL-6/STAT3 pathway in HCC cells (Fig. 2a, c) and DCA caused marked reduction of NDV-induced STAT3 activation (phosphorylated STAT3, p-STAT3) and *IL-6* expression (Fig. 2a, c). In addition, NDV infection markedly increased IDO1 expression in HCC cells at both protein and mRNA levels (Fig. 2a, b); this expression could be significantly attenuated by DCA treatment (Fig. 2a, b). Furthermore, p-STAT3 and IDO1 expression were markedly decreased by DCA even in PDK-1-silenced HCC cells with NDV infection (Fig. 2d), which excludes the possibility that DCA reduced p-STAT3 and IDO1 via PDK-1 inhibition. In an ascitic HCC mouse model (Fig. 1i), DCA decreased both NDV-induced p-STAT3 and IDO1 expression (Fig. 2e–g), and significantly reduced *Il-6* and *Ido1* mRNA levels in ascitic cells in vivo (Fig. 2h, i). Surprisingly, NDV treatment increased the number of MDSCs (CD11b^+^Gr-1^+^) in ascites; DCA could antagonise this effect (Fig. 2j, k). Taken together, these data suggest that DCA mitigates several critical immune negative feedback mechanisms, including STAT3 activation, IDO1 expression, and MDSC infiltration, in NDV-treated HCC.

### DCA promotes oncolytic NDV replication in HCC, both in vitro and in vivo

**Fig. 3:**
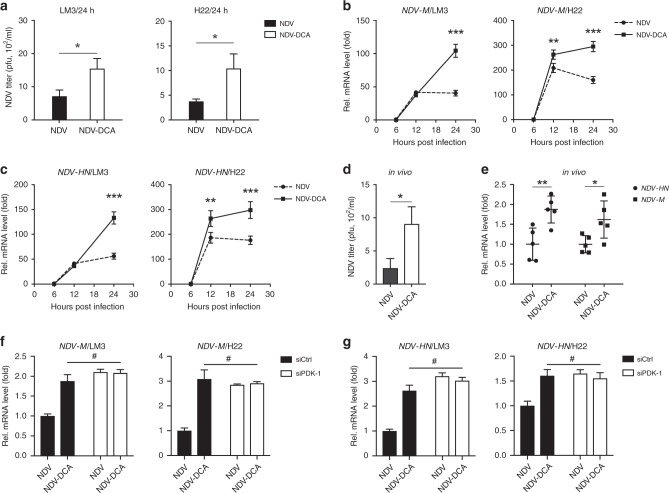
DCA promotes NDV replication in HCC in vitro and in vivo. **a** Cells were infected with NDV (MOI = 10) in the absence or presence of DCA (20 mM) for 24 h, then harvested; NDV titres were measured by plaque assay. Means and standard deviations of three independent experiments are shown. **b**, **c** LM3 and H22 cells were infected with NDV (MOI = 10) in the absence or presence of DCA (20 mM) for 6, 12, and 24 h, then harvested; *NDV-M* (**b**) and *NDV-HN* (**c**) gene expression levels were determined by qPCR. Means and standard deviations of quadruplicate samples are shown. **d**, **e** Mice were treated as previously described (Fig. 1I), ascitic cells were harvested on day 15, and the viral titres were determined by plaque assay (**d**); *NDV-HN* and *NDV-M* gene expression levels were quantified by qPCR (**e**); means and standard deviations of five mice are shown. **f**, **g** LM3 and H22 cells were transfected with human and mouse siRNA targeting PDK-1 for 4 h, respectively, followed by infected with NDV (MOI = 10) in the absence or presence of DCA (20 mM) for 24 h, then cells were harvested; *NDV-M* (**f**) and *NDV-HN* (**g**) gene expression levels were determined by qPCR. Means and standard deviations of quadruplicate samples are shown. *, *p* < 0.05; **, *p* < 0.01; ***, *p* < 0.001; ^#^, *p* > 0.05

We quantified the replication of NDV in HCC cells in the presence or absence of DCA. Interestingly, viral replication was significantly increased by DCA treatment in HCC cells, as shown by viral titres (Fig. 3a), expression of *NDV-HN* (hemagglutinin-neuraminidase), and *NDV-M* (matrix) genes (Fig. 3b, c). In HCC ascites (Fig. 1i), the viral titres (Fig. 3d), as well as the gene expression levels of both *NDV-HN* and *NDV-M* (Fig. 3e), were significantly increased by DCA treatment. We further determined whether DCA promoted viral replication via PDK-1 inhibition. Indeed, when PDK-1 was silenced by siRNA (Fig. S4), NDV replication could not be further increased by DCA in HCC cells (Fig. 3f, g). Taken together, these findings suggested that DCA increased NDV replication in a PDK-1-dependent manner in HCC cells.

### DCA enhances NDV-induced antitumour immune response in HCC

**Fig. 4:**
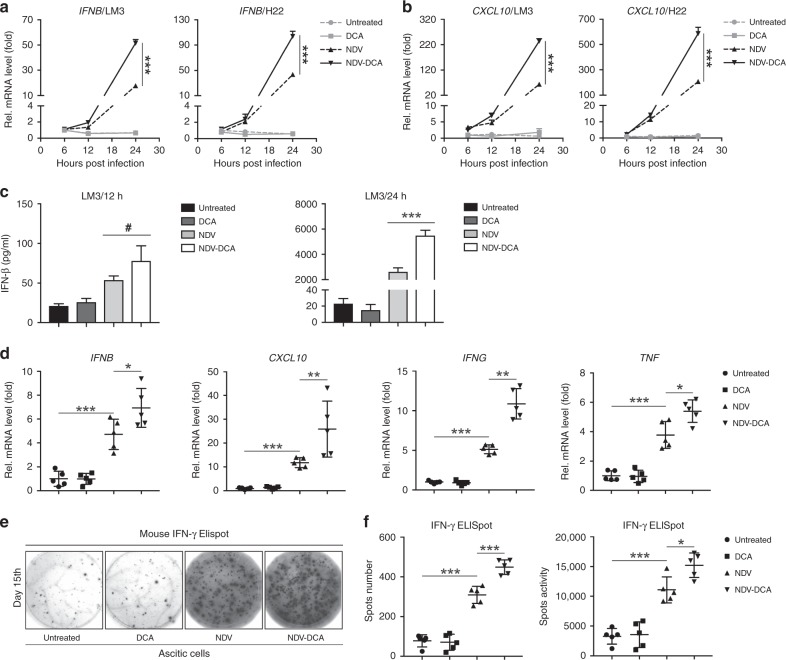
DCA improves NDV-induced antitumour immune responses in HCC. **a**, **b** LM3 and H22 cells were infected with NDV (MOI = 10) in the absence or presence of DCA (20 mM) for 6, 12, and 24 h, then harvested; *IFNB* (**a**) and *CXCL10* (**b**) gene expression levels were determined by qPCR. Means and standard deviations of quadruplicate samples are shown. **c** LM3 cells were infected with NDV (MOI = 10) in the absence or presence of DCA (20 mM) for 12 h and 24 h; cell culture media was then harvested, and IFN-β protein expression levels were determined by ELISA. Means and standard deviations of triplicate samples are shown. **d** Mice were treated as previously described (Fig. 1I), ascitic cells were harvested on day 15; *Ifnb, Cxcl10, Ifng*, and *Tnf* gene expression levels were quantified by qPCR. **e**, **f** Ascitic cells were harvested on day 15, then washed, counted, and analysed by IFN-γ ELISpot assay; representative graphs (**e**) and statistical analysis (**f**) are shown. Means and standard deviations of five mice are shown. *, *p* < 0.05; **, *p* < 0.01; ***, *p* < 0.001

We investigated whether DCA could enhance NDV-induced immune responses. We found that DCA markedly increased NDV-induced antiviral molecules, such as the gene expression of *IFNB* (Fig. 4a) and *CXCL10* (Fig. 4b), as well as IFN-β production (Fig. 4c). Similarly, gene expression levels of *IFNB, CXCL10, IFNG*, and *TNF* were significantly upregulated by NDV treatment and were further increased by DCA treatment in HCC ascites (Fig. 4d). Moreover, NDV significantly increased the number of IFN-γ producing cells in HCC ascites, and DCA could further enhance this effect (Fig. 4e, f). Taken together, these data indicated that DCA can strengthen oncolytic NDV-elicited immune activation in HCC.

### DCA improves NDV-mediated oncolysis and antitumour efficacy, and prolongs the lifespan of HCC-bearing mice

**Fig. 5:**
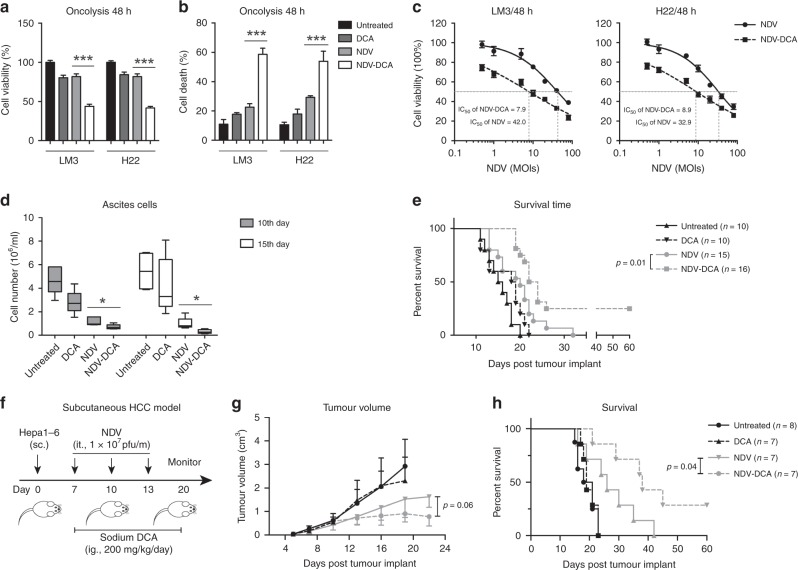
DCA enhances the NDV-mediated oncolysis and antitumour efficacy in HCC. **a**, **b** Cells were infected with NDV (MOI = 10) in the presence or absence of DCA (20 mM) for 48 h, and the oncolytic effects were determined by MTT assays (**a**) and trypan-blue exclusion assays (**b**). Means and standard deviations of quadruplicate samples are shown. **c** Cells were seeded in 96-well plates and infected with NDV at various MOIs (0, 1, 2, 5, 10, 20, 40, and 80) for 48 h, with or without DCA (20 mM). Cell viability was assessed by MTT assays. Means and standard deviations of quadruplicate samples and IC_50_ (half maximal inhibitory concentration) values of NDV are shown. **d**, **e** Mice were treated as previously described (Fig. 1I), ascitic cells were harvested after 10 or 15 days and cell numbers were determined by trypan-blue exclusion assays (**d**); means and standard deviations of five mice are shown. Kaplan–Meier survival curves were plotted (**e**) and analysed by log-rank (Mantel–Cox) test. All mice in each group were included in each analysis. **f**–**h** Mice were injected subcutaneously with 5 × 10^6^ Hepa1-6 cells. On days 7, 10, and 13, the mice received an intratumoural injection of 1 × 10^7^ pfu NDV, with or without DCA (200 mg/kg, daily, i.g.) (**f**). Tumour volumes were measured by calliper every 2–3 days before treatment; means and standard deviations of each group are shown (**g**). Kaplan–Meier survival curves were plotted (**h**) and analysed by log-rank (Mantel–Cox) test. All mice in each group were included in each analysis. *, *p* < 0.05; ***, *p* < 0.001

Finally, we evaluated the combinatorial oncolysis of NDV and DCA in HCC. DCA significantly enhanced the oncolytic efficacy of NDV in HCC cells, as determined by cell viability (Fig. 5a) and cell death (Fig. 5b). Consistent with this finding, DCA decreased the IC_50_ of NDV from MOI 42 to 7.9 in LM3 cells, and from MOI 32.9 to 8.9 in H22 cells, respectively (Fig. 5c). Furthermore, the therapeutic efficacy of NDV plus DCA was investigated in vivo. In a mouse ascitic HCC model (Fig. 1i), the combination of NDV and DCA resulted in a reduction of ascitic cells (Fig. 5d), which prolonged survival (25% of combination-treated mice survived for more than 60 days) (Fig. 5e). In a subcutaneous HCC model (Fig. 5f), DCA enhanced the therapeutic efficacy of NDV (Fig. 5g) and prolonged survival of the mice (Fig. 5h). Two of seven mice exhibited complete responses to combination treatment. No obvious therapy-associated side effects or bodyweight loss were recorded in either ascitic or subcutaneous HCC models (Fig. S5A, B). These results suggested that DCA improves the antitumour efficacy of NDV in HCC.

## Discussion

In the TME, lactate accumulation, STAT3 activation, IDO1 upregulation, and MDSC infiltration increase in a manner that shapes the immunosuppressive microenvironment. In the present study, while oncolytic NDV robustly activated immune responses in HCC, it simultaneously induced the aforementioned immunosuppressive factors. Interestingly, the PDK inhibitor, DCA, significantly reduced lactate release, STAT3 activation, IDO1 upregulation, and MDSC infiltration in NDV-treated HCC. Furthermore, DCA increased NDV replication in HCC. Consequently, DCA significantly enhanced antitumour immune responses, leading to improved antitumour efficacy and prolonged survival in HCC-bearing mice. Thus, combined therapy with DCA and NDV represents a promising oncolytic viro-immunotherapy.

It has been reported that lactate accumulation in the TME severely limits the functional properties of T cells and NK cells.^16,17^ Our data suggested that DCA has a larger effect on lactate formation than on glucose utilisation, which is consistent with enhancement of OXPHOS relative to glycolysis. It is plausible that DCA treatment might enhance the antitumour function of immune cells in the TME by reducing NDV-upregulated lactate production in HCC. Several studies have demonstrated that other OVs, such as adenovirus and measles virus, could upregulate aerobic glycolysis and lactate generation.^20,21^ Given the metabolic competition between cancer cells and immune cells in the TME,^15,33^ the suppression of glucose consumption by DCA might improve glucose availability to immune cells, in turn enhance immune cells proliferation and their effector function. In such circumstances, DCA treatment may support the ability of OVs to improve the antitumour immune responses.

NDV activates innate and adaptive immune factors, such as CD8^+^ T cells, NK cells, and type I IFNs, which exert critical antitumour effects;^12,13^ however, the immune negative regulators may concomitantly be upregulated. We found that STAT3 activation and IDO1 expression were markedly upregulated upon NDV infection. These regulators could suppress or tolerate CD8^+^ T and NK cell-mediated antitumour immune responses.^24^ Thus, they are likely to be detrimental to NDV-mediated viro-immunotherapy. In addition, a previous study suggested that STAT3 is an important player in the HIF-1a/PKM2/glycolysis feedback loop.^34^ It would be interesting to determine whether increased STAT3 activation by NDV is a mechanism by which NDV upregulates glycolysis in HCC.

Previous studies have reported that the ubiquitin–proteasome system participates in the degradation of IDO1^35^ and p-STAT3.^36^ However, we did not observe this phenomenon in DCA-treated HCC cells. In addition, we found that p-STAT3 and IDO1 expression were markedly decreased by DCA in PDK-1 silenced HCC cells with NDV infection, which excludes the possibility that DCA reduced p-STAT3 and IDO1 via PDK-1 inhibition. It has also been suggested that lactate is involved in STAT3 activation of macrophages in the TME.^37^ However, in the present study, we found that extracellular lactate did not activate the STAT3 signalling pathway in HCC cells, which excludes the possibility that DCA mitigates NDV-induced STAT3 activation by reducing lactate production. Further studies are needed to clarify the specific mechanisms by which DCA reduces p-STAT3 and IDO1.

We found that MDSCs were significantly increased in NDV-treated HCC. A previous study suggested that aerobic glycolysis signatures were correlated with high MDSC counts, low T-cell counts, and poor human triple-negative breast cancer outcome.^38^ Exogenous lactate has been shown to increase the generation of MDSCs from mouse bone marrow cells stimulated with GM-CSF and IL-6 in vitro.^17^ Knockdown of the key glycolytic enzyme, lactate dehydrogenase A, resulted in fewer MDSCs in tumour tissues and the spleen.^38^ It is plausible that DCA might reduce MDSC infiltration by targeting aerobic glycolysis in the TME after NDV treatment. In addition, the differentiation of MDSCs depends on STAT3 activation.^39^ The reduction in the number of MDSCs was partially related to reduced p-STAT3 levels. Furthermore, IDO1 was correlated with the expansion, recruitment, and activation of MDSCs in tumours;^40^ thus, the reduced expression of IDO1 following DCA treatment may have led to reduction of MDSC infiltration. These findings indicate that DCA may reduce MDSCs in multiple manners: by targeting aerobic glycolysis, and by inhibiting STAT3 activation and IDO1 expression.

Sufficient viral replication determines the therapeutic efficacy of oncolytic viro-immunotherapy. In addition to reducing immunosuppressive factors, DCA significantly enhanced NDV replication in HCC. This increased viral replication was dependent on PDK-1 expression. We found that antiviral innate immunity was not inhibited by DCA treatment in HCC cells. However, the viability of the host cells was maintained by DCA at the early stage of NDV infection (data not shown), which may have supported viral replication. Consistent with this hypothesis, our previous study showed that mitophagy enhanced the replication of NDV by inhibiting endogenous apoptosis in lung cancer cells.^41^ Further studies are needed to explore the relationship between PDK-1 expression and host cell viability during NDV infection.

In conclusion, DCA reduced STAT3 activation, IDO1 upregulation, and MDSC infiltration; it also promoted viral replication in HCC cells. These effects of DCA significantly improved NDV-mediated antitumour immunity and prolonged survival in mouse models of ascitic and subcutaneous HCC. These findings clarify the metabolic adaptation and immune negative feedback mechanisms during OV infection and provide a novel immune-metabolism therapeutic strategy that combines DCA with oncolytic NDV for HCC, which might be applied clinically in the near future.

## Supplementary information

Supplementary Information

## Data Availability

All data and materials generated during and/or analysed during the current study are available from the corresponding author on reasonable request.
