# Supplementary material for: Targeting aerobic glycolysis by dichloroacetate improves Newcastle disease virus-mediated viro-immunotherapy in hepatocellular carcinoma
Source: Br J Cancer. 2019 Dec 10;122(1):111–20. doi: 10.1038/s41416-019-0639-7 (PMC6964686; doi:10.1038/s41416-019-0639-7)
Supplement: Supplementary file 1 — Supplementary Information [file 41416_2019_639_MOESM1_ESM.docx]

**Supplementary Information**

**Supplementary figures and legends**

**Figure S1. The impact of DCA on the pH of the medium.**

The pH values of the DMEM medium containing various concentrations of DCA was detected by Mettler Toledo^TM^ FiveEasy Benchtop pH Meter.

**Figure S2. The expression of PDK subtypes in NDV-infected HCC cells.**

LM3 and H22 cells were infected with NDV at a MOI of 10 for 24 h, then cells were harvested, PDK-1, PDK-2, PDK-3 and PDK-4 mRNA expression levels were determined by qPCR. Means and standard deviations of quadruplicates are shown.

**Figure S3. The cytotoxicity and time kinetics of DCA and NDV in HCC cells.**

**(A, B)** LM3 and H22 cells were treated with DCA (A) or NDV (B) at indicated concentration for 48 h. Cell viability was measured by MTT assays. Means + SDs of quadruplicates and IC_50_ values of DCA or NDV are depicted. **(C, D)** Cells were treated with DCA (20 mM) (C) or NDV (MOI=10) (D) for 12, 24, 48 h, and cell viability was determined by MTT assays. Means + SDs of quadruplicates are shown.

**Figure S4. The silencing efficiency of PDK-1 siRNA in HCC cells.**

LM3 and H22 cells were transfected with PDK-1 siRNA for 4 h followed by infection with NDV (MOI = 10) in the presence or absence of DCA (20 mM) for another 24 h. Cells were harvested and PDK-1 mRNA expression level was determined by qPCR. Means and standard deviations of quadruplicates are shown.

**Figure S5. Combined therapy with NDV and DCA had no obvious side effects *in vivo*.**

**(A, B)** Mice received an ip. injection of 2 × 10^6^ H22 cells (A) or sc. injection of 5 × 10^6^ Hepa1-6 cells (B) followed by 1 × 10^7^ pfu NDV with or without DCA (200 mg/kg, daily, ig.) at the indicated time points (Fig. 5D, G). The body weight of mice was monitored and plotted. Means + SDs of all mice are shown.

**Supplementary methods**

**Table S1. Sequences of primers and siRNAs**

| **Primer sequences** | | | | |
| --- | --- | --- | --- | --- |
| Gene Name & NCBI Ref. Sequence | Forward (5’-3’) | Reverse (5’-3’) | Position | Length (bp) |
| *NDV-HN* (FJ004152.1) | GGGGGATAGGCAAAGAACTCATT | GTATTGGCCGTCGAACCCTAAC | 407-849 | 443 |
| *NDV-M* (AF099660.1) | AGTGATGTGCTCGGACCTTC | CCTGAGGAGAGGCATTTGCTA | 811-931 | 121 |
| Human *GAPDH* (NM_002046.6) | CCATGTTCGTCATGGGTGTGAACCA | GCCAGTAGAGGCAGGGATGATGTTC | 462-712 | 251 |
| Human *IFNB* (NM_002176.3) | CTTGGATTCCTACAAAGAAGC | CATCTCATAGATGGTCAATGC | 173-343 | 171 |
| Human *CXCL10* (NM_001565.3) | CTTCCAAGGATGGACCACACA | CCTTCCTACAGGAGTAGTAGCAG | 432-581 | 150 |
| Human *IL-6* (NM_000600.4) | GGTACATCCTCGACGGCATCT | GTGCCTCTTTGCTGCTTTCAC | 294-374 | 81 |
| Human *IDO1* (NM_002164.5) | GACGGTCTGGTGTATGAAG | ACTCTAATGAGCACAGGAAG | 847-1048 | 202 |
| Human PDK-1 (NM_002610.5) | CTGTGATACGGATCAGAAACCG | TCCACCAAACAATAAAGAGTGCT | 483-673 | 191 |
| Human PDK-2 (NM_002611.5) | GGGACCACAACCAAAGTCGC | GACATACCAGCTCTGCACCAG | 53-352 | 300 |
| Human PDK-3 (NM_005391.4) | TCAGGTGGTTTATGTGCCCTC | TAACCAAATCCAGCCAAAGGG | 925-1206 | 282 |
| Human PDK-4 (NM_002612.4) | ACAGACAGGAAACCCAAGCC | GTTGCCCGCATTGCATTCTTA | 772-1011 | 240 |
| Mouse *GAPDH* (NM_001289726.1) | TCTCCTGCGACTTCAACA | TGTAGCCGTATTCATTGTCA | 931-1047 | 117 |
| Mouse *IFNB* (NM_010510.1) | GTTCCTGCTGTGCTTCTC | TCTTCTCCGTCATCTCCATA | 50-219 | 170 |
| Mouse *CXCL10* (NM_021274.2) | ATGGATGGACAGCAGAGA | TAGAACTGACGAGCCTGA | 408-745 | 338 |
| Mouse *IL-6* (NM_031168.2) | CCACCAAGAACGATAGTCA | TTGTCACCAGCATCAGTC | 45-142 | 98 |
| Mouse *IDO1* (NM_008324.2) | GCAGTAGAGCGTCAAGAC | CCTCATACAGCAGACCTTC | 584-776 | 193 |
| Mouse *IFNG* (NM_008337.4) | CTTCTTCAGCAACAGCAAGG | TGAGCTCATTGAATGCTTGG | 412-512 | 101 |
| Mouse *TNF* (NM_013693.3) | ACGCTCTTCTGTCTACTGA | GCCATAGAACTGATGAGAGG | 303-391 | 89 |
| Mouse PDK-1 (NM_172665.5) | GCAAAGTTGGTATATCCAAAGCC | AGGAGTGCTGGTTGAGTAGC | 476-744 | 269 |
| Mouse PDK-2 (NM_133667.2) | GCACCCAAGTACATCGAGCA | TCTGGACATACCAGCTCTGC | 161-386 | 226 |
| Mouse PDK-3 (NM_145630.3) | TGCAGTCATTTATTTGAAGGCCC | ACGCGTCTCTGGTTGACTTG | 1257-1513 | 253 |
| Mouse PDK-4 (NM_013743.2) | TCCTGCCTGACCGCTTAGTG | GCGTGTCTACAAACTCTGACAGG | 337-474 | 138 |
| **Short interfering RNA sequences** | | | | |
| Targeting Gene & NCBI Ref. | Sense (5’-3’) | Anti-Sense (5’-3’) | Position | - |
| Human PDK-1 (NM_002610.4) | CCCGAACUAGAACUUGAAGAATT | UUCUUCAAGUUCUAGUUCGGGTT | 686-706 | - |
| Mouse PDK-1 (NM_172665.5) | GCAAAGUUGGUAUAUCCAATT | UUGGAUAUACCAACUUUGCTT | 3606-3624 | - |
| Non-Specific Control | UUCUCCGAACGUGUCACGUTT | ACGUGACACGUUCGGAGAATT | - | - |

Primers were synthesized by GeneScript, Inc. (Nanjing, China) and siRNAs were synthesized by GenePharma, Inc. (Shanghai, China)
